# Oncological Outcomes and Prognostic Factors in Soft Tissue Sarcoma of Children, Adolescents, and Young Adults: A Retrospective Single-Center Cohort Study

**DOI:** 10.3390/cancers18142328

**Published:** 2026-07-19

**Authors:** Nina Myline Engel, Jendrik Hardes, Evmorfia Pechlivanidou, Markus Nottrott, Dimosthenis Andreou, Luisa Kriens, Lars-Erik Podleska, Bosse Rüberg, Arne Streitbuerger, Rodanthi Margariti

**Affiliations:** 1 Department of Orthopedic Oncology, University Hospital Essen, 45147 Essen, Germany; ninamyline.engel@uk-essen.de (N.M.E.); markus.nottrott@uk-essen.de (M.N.); luisajosephine.kriens@uk-essen.de (L.K.);; 2 Faculty of Medicine, University of Bern, 3008 Bern, Switzerland; pevmorfia@med.uoa.gr

**Keywords:** soft tissue sarcoma, pediatric, adolescent and young adult, local recurrence, limb salvage, surgical margins

## Abstract

**Background:** Soft tissue sarcomas (STS) in children, adolescents, and young adults are rare and biologically heterogeneous, and prognostic factors for local recurrence, metastasis, and survival remain incompletely defined. This study aimed to characterize oncological outcomes and to explore clinical, surgical, and treatment-related factors associated with these endpoints at a tertiary sarcoma center. **Methods:** This retrospective single-center cohort study included 42 consecutive patients aged ≤25 years with histologically confirmed STS who underwent definitive surgery between June 2018 and November 2025. Local recurrence-free (LRFS), metastasis-free (MFS), event-free (EFS), and overall survival (OS) were estimated by the Kaplan–Meier method with competing-risks sensitivity analyses. Given the limited number of events, pre-specified univariable comparisons (log-rank, Fisher exact, Mann–Whitney U) were performed and regarded as exploratory. **Results:** The median age was 16.7 years and 26 patients (61.9%) were female. A microscopically complete (R0) resection was achieved in 35 of 41 patients with assessable margins (85.4%), and limb preservation in 38 (90.5%). Over a median follow-up of 23.1 months, the 24- and 60-month estimates were LRFS 85.7% and 79.6%, MFS 93.3% and 84.8%, EFS 82.3% and 64.7%, and OS 96.0% and 81.5%, respectively. Metastatic disease at diagnosis was the only variable associated with metastatic status at last follow-up (*p* = 0.035). No factor was robustly associated with local recurrence; anatomical site (*p* = 0.68), resection-margin status (*p* = 0.23), and tumor volume (*p* = 0.97) were not significant, and an apparent association with a sub-millimeter margin rested on a single event (complete separation). **Conclusions:** Metastatic disease at diagnosis emerged as the most important prognostic factor for MFS in our cohort. Despite the complexity of treatment in this young patient population, high rates of complete resection and limb preservation were achieved, highlighting the value of multidisciplinary management in specialized sarcoma centers. While the limited cohort size did not allow the identification of further statistically significant prognostic factors for local recurrence, such associations may become detectable in larger multicenter studies.

## Simple Summary

Soft tissue sarcomas are rare cancers that arise in muscle, fat, and connective tissue. When they occur in children, adolescents, and young adults they are difficult to treat, because they are uncommon, biologically diverse, and often located close to nerves, blood vessels, and growing bone. It is still unclear which of these young patients are most likely to have their tumor return at the same site or spread to distant organs. We reviewed 42 consecutive patients aged 25 years or younger whose sarcoma was removed at a specialized sarcoma center between 2018 and 2025. The tumor was removed completely, with no cancer cells at the cut edges, in 85% of patients, and the affected limb could be preserved in 90%. The only factor clearly linked to outcome was whether the disease had already spread at the time of diagnosis. Neither the site of the tumor, nor its size, nor the width of the surgical margin predicted local recurrence, most probably because the number of patients and of events was small. Our findings support treatment in specialized centers and show why larger, multicenter studies are needed to identify reliable risk factors in this age group.

## 1. Introduction

Soft tissue sarcomas (STS) occur across all age groups, although their incidence and histopathological distribution differ markedly between pediatric and adult patients [1]. They account for approximately 7–8% of all childhood malignancies [2]. In younger patients, the most common histological subtypes include rhabdomyosarcoma (RMS), synovial sarcoma (SS), clear cell sarcoma (CCS), and alveolar soft part sarcoma (ASPS), whereas undifferentiated pleomorphic sarcoma (UPS), liposarcoma (LS), and myxofibrosarcoma (MFS) predominate in adults. This distinction is not merely descriptive. The current World Health Organization classification of soft tissue tumors defines entities increasingly on the basis of their molecular alterations, and several of the subtypes that cluster in childhood and adolescence are characterized by recurrent gene fusions rather than by the complex genomic profiles typical of adult pleomorphic sarcomas [3]. Regardless of age, the management of these predominantly high-grade malignancies is typically multidisciplinary, with complete surgical resection central to local control and radiotherapy and chemotherapy used in neoadjuvant or adjuvant settings depending on histology, tumor extent, and risk profile [4]. A further layer of complexity arises from the fact that rhabdomyosarcoma and non-rhabdomyosarcoma soft tissue sarcoma (NRSTS) are managed according to separate protocols, staging conventions, and risk-stratification systems, so that patients of similar age treated at the same institution may follow substantially different therapeutic pathways [4].

Identifying prognostic factors for metastatic progression and local recurrence is important for risk stratification, treatment planning, and follow-up scheduling. Previous studies have shown that tumor grade, tumor size, resectability, initial tumor stage, and prior incomplete excision may be associated with unfavorable outcomes, and these factors underpin contemporary risk-stratified treatment strategies [5]. Surgical management is a further determinant of outcome: nationwide data in adults indicate that patients with deep-seated, non-low-grade tumors resected in high-volume hospitals achieve better long-term survival than those operated on in low-volume hospitals, an argument for centralized care that is at least as compelling in the pediatric and adolescent setting, where smaller anatomical dimensions and growth-related considerations add to the surgical complexity [6]. However, pediatric STS are rare, and the available evidence remains limited by small sample sizes, histological heterogeneity, and predominantly retrospective cohort data. Adolescents and young adults are additionally underrepresented in both pediatric and adult clinical trials and are distributed across pediatric and adult treatment networks, which fragments the evidence base further. As a result, prognostic factors for metastatic progression, local control, and survival remain incompletely defined in pediatric and adolescent populations, and contemporary single-center series that report surgical and pathological detail alongside oncological endpoints continue to contribute data that larger registries do not capture.

The objective of this retrospective single-center study was to characterize oncological outcomes and to explore the clinical, surgical, and treatment-related factors associated with metastatic progression, local recurrence, and survival in children, adolescents, and young adults with soft tissue sarcoma treated at a specialized tertiary sarcoma center. A secondary aim was to describe the surgical burden of treatment in this age group—including limb preservation, resection of adjacent bone, vascular reconstruction, plastic soft tissue coverage, and amputation—and to delineate transparently what a cohort of this size can and cannot establish.

## 2. Materials and Methods

This retrospective single-center cohort study included all consecutive children, adolescents, and young adults (≤25 years) with histologically confirmed soft tissue sarcoma who underwent definitive surgical treatment at a tertiary referral sarcoma center between June 2018 and November 2025. Patients without histological confirmation or without definitive surgical treatment at our institution were excluded. In total, 42 patients were included. The study was approved by the local ethics committee (24-12119-BO). The report follows the Strengthening the Reporting of Observational Studies in Epidemiology (STROBE) recommendations for cohort studies [7].

Demographic, clinical, pathological, treatment, and follow-up data were retrospectively extracted from the institutional hospital information system and electronic medical records. Histopathological reports, operative notes, imaging studies, and oncological follow-up data were reviewed for all included patients. Tumor volume was calculated from preoperative magnetic resonance imaging using the ellipsoid formula (length × width × height × π/6) and expressed in cubic centimeters. Surgical margins were classified by histopathological assessment as microscopically negative (R0) or positive (R1); for R0 resections, the closest microscopic tumor-to-margin distance was recorded and categorized as <1 mm, 1–5 mm, or >5 mm.

As no R2 or Rx resections occurred in this cohort, these categories were not included in the subsequent classification and analyses.

### Statistical Analysis

Continuous variables are summarized as medians with interquartile ranges (IQR) and categorical variables as frequencies and percentages; exact (Clopper–Pearson) 95% confidence intervals (CIs) were calculated for principal proportions. The index date for all time-to-event analyses was the date of the definitive tumor resection performed at our institution. In patients referred after a local recurrence that had been managed elsewhere—in whom the institutional procedure constituted the resection of that recurrence—this operation was used as the index event, and the pre-referral recurrence was not counted as a local failure.

Local recurrence-free survival (LRFS) was measured from the index resection to the first local recurrence; metastasis-free survival (MFS) was assessed in patients with localized disease at diagnosis and measured to the first distant metastasis; overall survival (OS) was measured to death from any cause; and event-free survival (EFS) was defined as the time to the first of local recurrence, distant metastasis, or death. Distributions were estimated by the Kaplan–Meier method (deaths censored for LRFS and MFS), and 24- and 60-month estimates are reported with Greenwood 95% CIs. Because the complement of the Kaplan–Meier estimator overstates the crude incidence of an event when a competing event is present, cumulative incidences were additionally estimated within a competing-risks framework using the Aalen–Johansen estimator, with death treated as the competing event; the two sets of estimates are reported alongside each other so that the influence of competing mortality can be judged directly [8]. The median follow-up of survivors was estimated by the reverse Kaplan–Meier method [9]. Time-to-event analyses of local recurrence, metastasis, and events (LRFS, MFS, EFS) were restricted to patients who contributed post-index follow-up time (n = 37), whereas vital status for overall survival was ascertained for all 42 patients.

Owing to the small number of events, multivariable modeling was not appropriate and was not undertaken; prognostic analyses were restricted to pre-specified univariable comparisons and regarded as exploratory. With five local recurrences, two metastatic events among patients with localized disease at diagnosis, three deaths, and eight composite events, the number of events available per candidate variable lay between two and eight for every endpoint. This is well below the conventional threshold of approximately ten events per variable, below which regression coefficients become biased in both directions, large-sample variance estimates become unreliable, and confidence intervals lose their nominal coverage [10]. The threshold has been shown to be conservative under favorable conditions, but the proposed relaxations apply to settings with substantially more events than were available here and do not extend to cohorts in which entire comparator strata contain no events [11]. We therefore refrained deliberately from fitting Cox or logistic models, from constructing a risk score or nomogram, and from internal or external validation of any such model, since these analyses would have conveyed a precision that the underlying data cannot support. Subgroups were compared with the log-rank test, categorical associations with Fisher’s exact test, and continuous variables with the Mann–Whitney U test. No adjustment for multiple comparisons was applied; given the exploratory intent, *p* values are reported descriptively and are not intended to support confirmatory inference. Where a comparator stratum contained no events, proportional-hazards estimation did not converge (complete separation), and only the descriptive result is reported. All tests were two-sided, and *p* < 0.05 was considered significant. Analyses were performed in Python 3.12 (pandas v2.2, lifelines v0.28, SciPy v1.13, and NumPy v1.26).

## 3. Results

### 3.1. Patient and Tumor Characteristics

Forty-two patients were included (26 female, 61.9%; median age 16.7 years, IQR 13.2–19.6; median tumor volume 46.0 cm^3^, IQR 10.2–110.0). The lower extremity and limb girdle were most frequently involved (28 patients, 66.7%), followed by the upper extremity and the axial/trunk region (7 each). Synovial sarcoma (n = 9) and rhabdomyosarcoma (n = 7) were the predominant histologies, followed by fibroblastic/fibrosarcomatous tumors (n = 6) and undifferentiated pleomorphic sarcoma (n = 4). Seven of 40 patients with documented staging (17.5%) had metastatic disease at presentation, and eight (19.0%) had undergone an unplanned excision before referral. Neoadjuvant chemotherapy was administered to 26 patients (61.9%) and neoadjuvant radiotherapy to eight (19.0%). A microscopically complete resection (R0) was achieved in 35 of 41 patients with assessable margins (85.4%)—with the closest margin <1 mm in four, 1–5 mm in 20, and >5 mm in eight—whereas five patients had an R1 resection and margins were not assessable in one. Of patients with histopathologically confirmed R1 margins, two had undergone previous incomplete external tumor resection, while one patient underwent surgery in a palliative setting. Limb preservation was achieved in 90.5% of patients at the last follow-up. Major amputation was required in four cases: one primary amputation due to extensive initial tumor involvement and three secondary amputations (local tumor progression in one patient two years after initial resection; radiation-induced secondary sarcoma in two patients, occurring at a mean interval of 7.7 years after initial sarcoma treatment including radiotherapy). The median follow-up was 23.1 months (Table 1).

Four patients required plastic reconstruction for soft tissue defect coverage during primary or complications treatment. Overall, wound healing complications occurred in four patients, necessitating surgical treatment. No intraoperative complications were observed.

A vascular graft was required in three patients to preserve the limb during primary surgery. In patients undergoing limb-sparing procedures, partial resection of adjacent bone was performed in seven cases. The resulting bony defect required reconstruction or stabilization in two of these patients (Palacos spacer, n = 1; tumor prosthesis, n = 1).

### 3.2. Local Control

**Figure 1:**
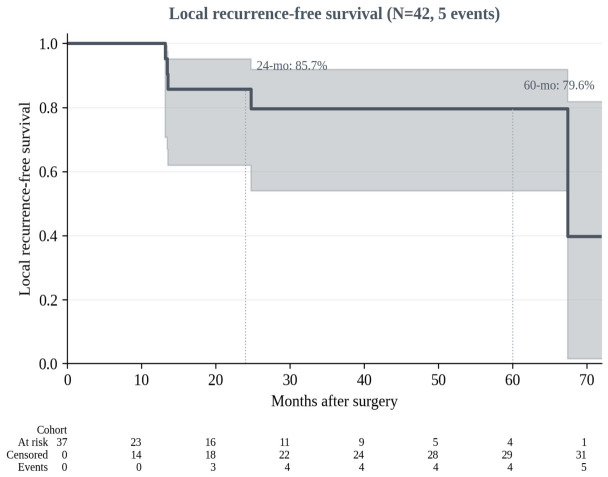
Kaplan–Meier estimate of local recurrence-free survival with 95% confidence band and numbers at risk (5 events among 37 evaluable patients).

Five local recurrences occurred, at 13.2, 13.5, 13.5, 24.8, and 67.4 months after the index resection. Estimated LRFS was 85.7% (95% CI 62.0–95.2) at 24 months and 79.6% (95% CI 53.9–91.9) at 60 months (Figure 1); the competing-risks cumulative incidence of local recurrence was 13.8% and 19.7%, respectively, indicating minimal influence of competing mortality.

All five recurrences followed an R0 resection; none occurred among the five patients with R1 margins. This counterintuitive pattern is most plausibly explained by the very small R1 subgroup (n = 5) and short follow-up, and should not be interpreted as evidence that positive margins confer a lower recurrence risk. The recurrences spanned the full range of margin widths, including one tumor resected with a margin > 5 mm, and resection-margin status (R0 vs. R1) was not associated with local recurrence (*p* = 0.23). A closest margin < 1 mm appeared associated with recurrence on the log-rank test, but this comparison involved only four patients and a single event, produced complete separation, and could not be stably estimated; it should be regarded as hypothesis-generating only. Combining R1 resections and R0 resections < 1 mm into an “inadequate-margin” category did not identify a higher-risk group (*p* = 0.55). Anatomical site (*p* = 0.68), tumor volume (*p* = 0.97), and prior unplanned excision (*p* = 0.55) were not associated with local recurrence. All five recurrences occurred in patients who had received neoadjuvant chemotherapy (none of the 16 chemotherapy-naïve patients recurred); this complete separation precluded stable estimation, and the apparent associations of neoadjuvant treatment with inferior local control most plausibly reflect confounding by indication rather than a deleterious effect. Overall, no examined variable reliably predicted local recurrence.

### 3.3. Distant Control

**Figure 2:**
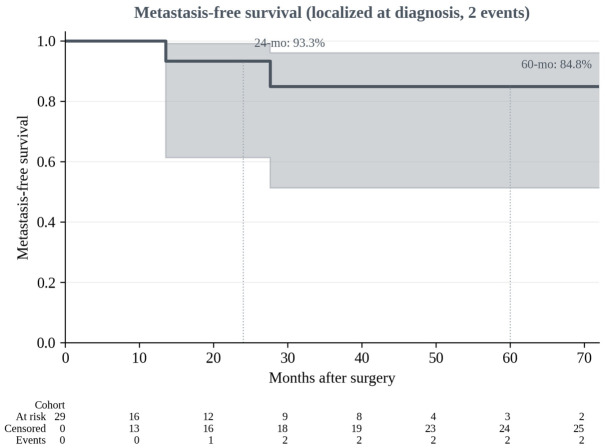
Kaplan–Meier estimate of metastasis-free survival in patients with localized disease at diagnosis (2 events).

Of the 40 patients with documented staging, 33 had localized disease at diagnosis; 31 of these were evaluable for metastasis-free survival, of whom 29 contributed follow-up time. Two developed distant metastases; estimated MFS was 93.3% (95% CI 61.3–99.0) at 24 months and 84.8% (95% CI 51.2–96.0) at 60 months (Figure 2). Metastatic disease at diagnosis was the only variable associated with metastatic status at last follow-up (Fisher exact test, *p* = 0.035); tumor volume (*p* = 0.44), age (*p* = 0.82), and margin status (*p* = 1.0) were not associated. Among the seven patients with metastatic disease at presentation, five had persistent or progressive metastatic disease at last follow-up. This association reflects the expected persistence of disease among patients who already had metastatic involvement at presentation rather than an independently identified prognostic factor, and should be interpreted accordingly.

### 3.4. Overall and Event-Free Survival

**Figure 3:**
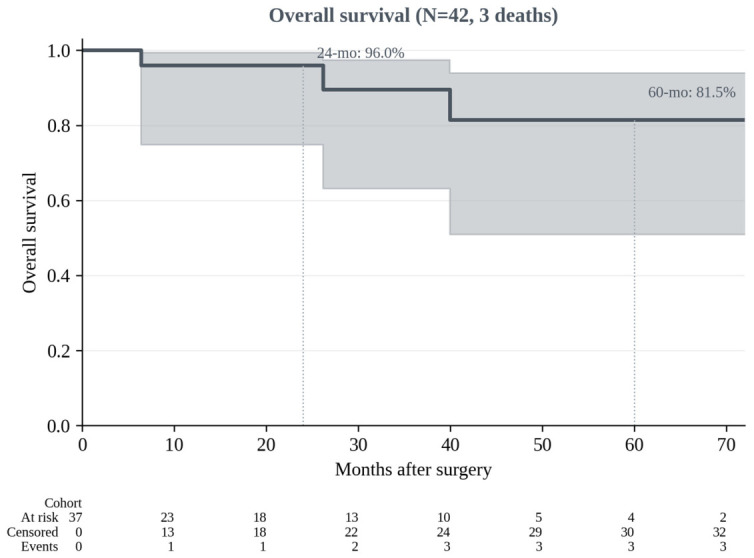
Kaplan–Meier estimate of overall survival with 95% confidence band and numbers at risk (3 events).

**Figure 4:**
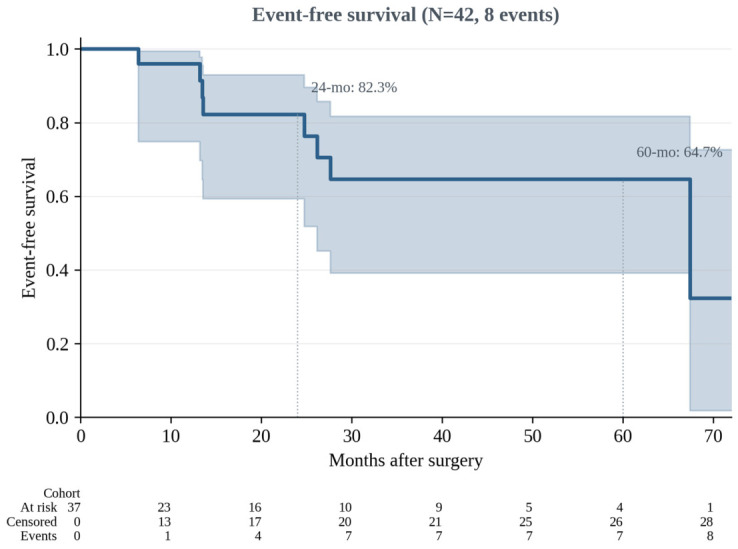
Kaplan–Meier estimate of event-free survival (first of local recurrence, distant metastasis, or death; 8 events).

**Figure 5:**
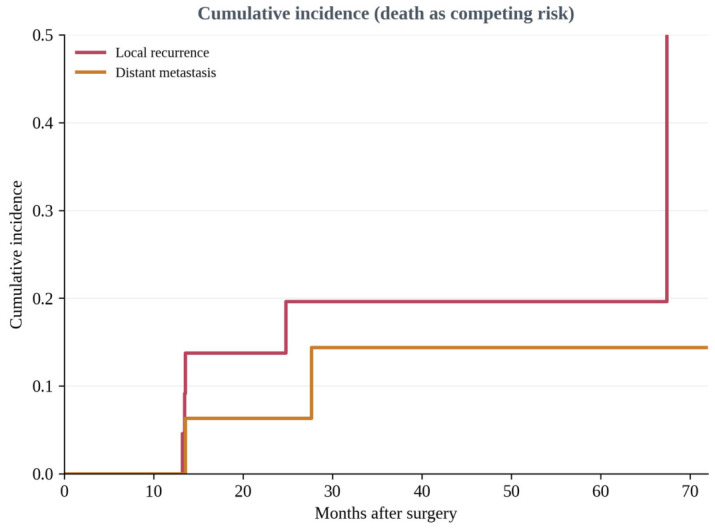
Cumulative incidence of local recurrence and of distant metastasis estimated within a competing-risks framework, with death treated as a competing event.

Three patients died, at 6.4, 26.1, and 39.9 months. Estimated OS was 96.0% (95% CI 74.8–99.4) at 24 months and 81.5% (95% CI 50.9–94.0) at 60 months (Figure 3). With only three deaths the study had no power to identify prognostic factors for survival; any apparent association with neoadjuvant radiotherapy was generated by a single death in the irradiated subgroup and reflects confounding by treatment indication rather than a treatment effect (exploratory comparisons for survival are not reported given the small number of deaths). Considering local recurrence, distant metastasis, and death together, eight composite events were observed, with EFS of 82.3% (95% CI 59.3–93.0) at 24 months and 64.7% (95% CI 39.1–81.7) at 60 months (Figure 4). Competing-risks cumulative incidences of local recurrence and distant metastasis (Figure 5) closely matched the complementary Kaplan–Meier estimates, confirming that the few competing deaths did not materially bias the cause-specific analyses (Table 2).

### 3.5. Five-Year Survival Estimates

Projected five-year outcomes were 79.6% for LRFS, 84.8% for MFS, 64.7% for EFS, and 81.5% for OS (Table 3). Because only three to four patients remained at risk at 60 months, these 60-month estimates are exploratory and are provided for descriptive completeness only; they should not be used for individual patient counseling or cross-study comparison. These estimates should be interpreted as preliminary: the median follow-up was 23.1 months, only three to four patients remained at risk at 60 months, and the confidence intervals are correspondingly wide. The small number of events precluded development of a multivariable prognostic or risk-prediction model; the five-year figures are best read as a benchmark for this rare population, to be refined as the cohort matures.

## 4. Discussion

In this retrospective cohort of children, adolescents, and young adults with soft tissue sarcoma treated at a specialized tertiary center, metastatic disease at diagnosis was the only factor consistently associated with oncological outcome. Patients presenting with metastatic disease were more likely to demonstrate persistent or progressive metastatic disease during follow-up, underscoring the importance of initial disease stage as a determinant of prognosis. These findings are consistent with previous reports identifying metastatic disease at presentation as one of the strongest adverse prognostic factors in pediatric STS. Sawamura et al. similarly demonstrated inferior outcomes in patients presenting with metastatic disease [12], and larger contemporary series have confirmed the central role of disease stage in risk stratification [13]. It should be acknowledged that this association is, in part, a restatement of the natural history of disseminated disease rather than the discovery of an independent prognostic factor: patients who present with metastases are, unsurprisingly, those most likely to still have metastases later. Its practical value lies elsewhere—in reinforcing the need for accurate staging at diagnosis, for stage-adapted intensity of systemic therapy, and for closer surveillance of patients with disseminated disease.

The interpretation of treatment-related variables warrants particular caution. In our cohort, all local recurrences occurred in patients who had received neoadjuvant chemotherapy, and univariable analyses suggested inferior local control among patients treated with neoadjuvant modalities. These findings should not be interpreted as evidence of treatment-related harm: neoadjuvant chemotherapy and radiotherapy are preferentially administered to patients with larger tumors, anatomically challenging lesions, high-grade histologies, or metastatic disease, so that the observed associations most likely reflect confounding by indication. The same caution applies to the nominal association between neoadjuvant radiotherapy and overall survival, which was generated by a single death in the irradiated subgroup.

Eight patients (19.0%) in our cohort had undergone an unplanned excision before referral, a proportion consistent with the referral pattern of a tertiary sarcoma center, and prior unplanned excision was not associated with local recurrence in our data (*p* = 0.55). This should not be read as reassurance. A systematic review and meta-analysis of re-excision after unplanned excision found that residual tumor in the re-resection specimen was associated with an approximately threefold increase in the odds of local recurrence, of distant metastasis, and of reduced overall survival, whereas patients who underwent systematic re-excision achieved oncological outcomes comparable with those of patients treated by planned resection from the outset [14]. The current debate concerns whom to re-excise rather than whether re-excision is effective. A large single-center series reported no significant difference in overall survival or local recurrence between patients who underwent re-resection and those managed conservatively, with the disadvantage confined to the subgroup in which residual disease was actually found, and argued for a more selective approach in low-grade lesions [15]. Our null result is compatible with either position and, with five events in total, discriminates between neither. It does, however, underline the practical point that the eight affected patients required an additional operation, with the attendant morbidity, for a lesion that had already been violated.

In contrast to several adult and larger pediatric series, anatomical location, tumor size, age, and prior unplanned excision were not significantly associated with local recurrence or metastatic progression, and neither resection-margin status nor margin distance demonstrated a robust association with local control [16,17,18,19]. These discrepancies are most plausibly explained by the limited sample size, the small number of outcome events, and the marked biological heterogeneity of pediatric STS. They must not be read as evidence that complete excision is unimportant; on the contrary, the cohort was underpowered to detect such effects, and the sub-millimeter-margin comparison produced complete separation that precluded estimation. In the prospective ARST0332 study, high-grade NRSTS achieved excellent local control after R0 resection, with lower control after R1 resection, reinforcing the central role of complete excision [20]. To the best of our knowledge, few studies have stratified resection margins by millimeter-defined thresholds specifically in pediatric and young adult soft tissue sarcoma patients. However, margin assessment should not be limited to millimeter-defined thresholds alone [21]. Future structured analyses should extend beyond margin width in millimeters to incorporate tumor biology, the influence of neoadjuvant therapy, biological barrier tissues, and the distinction between planned and unplanned close margins; the role of routine re-excision after an unplanned resection likewise remains debated [22]. Complete resection at a specialized sarcoma center remains the surgical gold standard [23], and resection is often more demanding in children than in adults because of smaller anatomical dimensions and the characteristics of the growing organism [24]. Pathological response to neoadjuvant therapy (tumor vitality) was recorded but, given incomplete documentation and the small number of events, was not formally analyzed; its prognostic relevance warrants evaluation in larger cohorts.

Synovial sarcoma was the most common subtype in our cohort, which is consistent with the histological distribution reported for adolescents and young adults and differs from the pattern seen in younger children, in whom rhabdomyosarcoma predominates. In children, adolescents, and young adults this entity generally carries a comparatively favorable prognosis, and surgery alone may be sufficient for low-risk, completely resected tumors, whereas multimodal therapy is reserved for larger or higher-risk disease [25,26]. The predominance of this comparatively favorable histology, together with the high proportion of extremity tumors, should be borne in mind when the survival estimates reported here are compared with those of series in which axial and head-and-neck primaries or fusion-positive rhabdomyosarcoma are more heavily represented.

Limb preservation was achieved in 90.5% of our patients, and the four amputations in this cohort illustrate the range of indications that persist despite modern multimodal therapy: one primary amputation for extensive initial tumor involvement, one secondary amputation for local progression two years after the initial resection, and two secondary amputations for radiation-induced secondary sarcoma arising at a mean interval of 7.7 years after the initial sarcoma treatment. The latter deserve emphasis. Long-term follow-up of childhood cancer survivors has established that survivors of a sarcoma who received radiotherapy are among those at highest risk of a subsequent malignant neoplasm, and that this risk remains elevated more than twenty years after the primary diagnosis [27]. In parallel, data from the Childhood Cancer Survivor Study indicate that amputation after primary limb salvage is not merely an early complication but a cumulative long-term risk, reaching 18% at twenty-five years after diagnosis, and that patients who ultimately undergo amputation report poorer physical health-related quality of life than those whose limb is preserved [28]. A limb preservation rate measured at a median follow-up of 23.1 months, as in the present cohort, therefore describes an early result rather than a definitive one. Both observations argue for structured, lifelong surveillance of these patients rather than for discharge after five event-free years, and they place the two radiation-associated sarcomas in our series in a context that a short-term local-control figure alone would obscure.

The five-year overall survival of 81.5% across all ages and histologies may be considered favorable and is in line with the literature, although the small number of patients still at risk at that time point requires that the figure be read as a benchmark rather than as a precise estimate. Harrison et al. reported a five-year overall survival of 52% in adolescents and young adults versus 78% in children with rhabdomyosarcoma (*p* < 0.001) and identified tumor site, size, invasiveness, clinical group, and histological subtype as prognostic among AYA patients; they also noted distinct metastatic patterns, with pulmonary metastases predominating in children and bone-marrow and lymph-node involvement more common in AYA patients—differences relevant both at diagnosis and during follow-up [13]. With increasing use of molecular pathological classification, more precise diagnostic stratification and prognostic information are becoming available. Fusion status in rhabdomyosarcoma is the clearest example: in a prospective Children’s Oncology Group analysis, patients with PAX3–FOXO1 or PAX7–FOXO1 fusion-positive alveolar tumors had significantly worse event-free survival than those with embryonal tumors, whereas fusion-negative alveolar tumors behaved like embryonal disease—a finding that has since been incorporated into risk stratification and treatment allocation [29]. Molecular characterization is also increasingly therapeutic rather than merely descriptive: larotrectinib produced durable responses across seventeen tumor types in adults and children whose tumors harbored an NTRK gene fusion, irrespective of histology or patient age [30]. Such developments make it likely that the prognostic architecture of this disease will be defined by biology at least as much as by the clinical and surgical variables examined here [31]. Tissue should therefore undergo standardized conventional and molecular pathological assessment, ideally within multicenter study protocols [32].

Several limitations should be acknowledged. The retrospective design introduces the possibility of selection and information bias. The cohort size was limited by the rarity of pediatric STS, yielding few outcome events—particularly for metastatic progression and death—so that multivariable analyses were not appropriate and all prognostic findings are exploratory; the cohort could not support a multivariable prognostic model or nomogram, tools of increasing value in soft tissue sarcoma [33]. The cohort was histologically heterogeneous, reflecting real-world practice but potentially obscuring subtype-specific associations. In particular, rhabdomyosarcoma and non-rhabdomyosarcoma soft tissue sarcomas differ in biology, staging systems, and treatment paradigms; pooling them may mask subtype-specific effects, and subtype-stratified survival analyses were precluded by the very small number of events per histological subtype. The median follow-up of 23.1 months is short relative to the natural history of several of the entities represented, and the 60-month estimates rest on three to four patients at risk. Finally, comprehensive molecular data were unavailable for the earliest patients.

A further consideration concerns what was deliberately not done. With between two and eight events per endpoint, multivariable adjustment, machine-learning enrichment, and internal or external validation of a prognostic model were all technically feasible, but they would have been methodologically indefensible: the resulting estimates would have appeared precise while in fact being driven by modeling assumptions rather than by the data [10,11]. We have therefore reported unadjusted estimates together with their full uncertainty, named the instances of complete separation explicitly rather than omitting the affected comparisons, and presented the counterintuitive margin findings as they were observed rather than reframing them. Despite these limitations, the study provides contemporary, detailed surgical, pathological, and oncological data from a specialized center, and the use of competing-risks and reverse Kaplan–Meier methods strengthens the reported outcome measures. We regard a small, transparently characterized cohort whose numbers can be pooled in future multicenter analyses as the more useful contribution to a disease of this rarity.

## 5. Conclusions

Metastatic disease at diagnosis was the only factor consistently associated with oncological outcome in this cohort. By contrast, no clinical, surgical, or treatment-related factor—including anatomical location and resection-margin status—was robustly associated with local control, although the small number of events limits firm conclusions. The high rate of microscopically complete resection and limb preservation in 90.5% of patients underscores the value of centralized, multidisciplinary management of young patients with soft tissue sarcoma and highlights the need for larger multicenter studies to refine risk stratification and treatment, particularly in the context of molecular pathological assessment and the resulting opportunities for targeted therapy.

## Figures and Tables

**Table 1:** Patient and tumor characteristics.

Characteristic	Value (N = 42)
Female sex	26 (61.9%)
Age at surgery, years—median (IQR)	16.7 (13.2–19.6)
Tumor volume, cm^3^—median (IQR) ᵃ	46.0 (10.2–110.0)
Lower extremity/limb girdle	28 (66.7%)
Upper extremity	7 (16.7%)
Axial/trunk	7 (16.7%)
Synovial sarcoma	9 (21.4%)
Rhabdomyosarcoma	7 (16.7%)
Fibroblastic/fibrosarcoma	6 (14.3%)
Undifferentiated pleomorphic sarcoma	4 (9.5%)
Other histology	16 (38.1%)
Metastatic at diagnosis	7/40 (17.5%; 95% CI 7.3–32.8)
Prior unplanned (“whoops”) excision	8 (19.0%)
Neoadjuvant chemotherapy	26 (61.9%)
Neoadjuvant radiotherapy	8 (19.0%)
Definitive resection R0	35/41 (85.4%; 95% CI 70.8–94.4)
margin <1 mm/1–5 mm/>5 mm	4/20/8
Definitive resection R1	5
Limb preservation	38 (90.5%; 95% CI 77.4–97.3)

ᵃ Available in 38 patients. CI, confidence interval (exact, Clopper–Pearson).

**Table 2:** Survival estimates at 24 and 60 months.

Endpoint	Events/n	24-Month % (95% CI)	60-Month % (95% CI)
Local recurrence-free survival	5/37	85.7 (62.0–95.2)	79.6 (53.9–91.9)
Metastasis-free survival ᵇ	2/29	93.3 (61.3–99.0)	84.8 (51.2–96.0)
Event-free survival	8/37	82.3 (59.3–93.0)	64.7 (39.1–81.7)
Overall survival	3/42	96.0 (74.8–99.4)	81.5 (50.9–94.0)

ᵇ Assessed in patients with localized disease at diagnosis (33 localized; 31 evaluable, of whom 29 contributed follow-up time). Deaths censored; competing-risks analyses gave concordant estimates.

**Table 3:** Projected five-year survival estimates with numbers at risk.

Endpoint	5-Year Estimate (95% CI)	No. at Risk at 60 Mo
Local recurrence-free survival	79.6% (53.9–91.9)	4
Metastasis-free survival	84.8% (51.2–96.0)	3
Event-free survival	64.7% (39.1–81.7)	4
Overall survival	81.5% (50.9–94.0)	4

## Data Availability

The data that support the findings of this study, together with the analysis code, are available from the corresponding author upon reasonable request.
